# Toward Precision Mental Health: Explainable Machine Learning Models for Screening Anxiety and Depression in Medical Students Based on Lifestyle and Socio-Demographic Factors–Pakistan

**DOI:** 10.1192/bjo.2026.11178

**Published:** 2026-06-30

**Authors:** Farah Rashid, Ahmed Waqas, Rafay Siddiqui, Talha Ahmed, Atif Rahman

**Affiliations:** 1 University of Liverpool, Liverpool, United Kingdom; 2 Health Services Academy, Islamabad, Pakistan; 3 National University of Sciences and Technology, Islamabad, Pakistan; 4 Rheinland-Pfälzische Technische Universität Kaiserslautern, Kaiserslautern, Germany; 5 California State Polytechnic University, Pomona, California, USA

## Abstract

**Aims:**

Anxiety and depression are prevalent among medical students worldwide, especially in low- and middle-income countries like Pakistan, where mental health stigma remains a significant challenge. This study aimed to develop an explainable machine learning-based screening tool for anxiety and depression using non-stigmatizing lifestyle and sociodemographic factors in academic settings. We hypothesized that explainable machine learning models can use non-stigmatizing lifestyle and sociodemographic factors to screen for anxiety and depression in medical students.

**Methods:**

A cross-sectional survey was conducted among 1,630 undergraduate medical students in Islamabad, Pakistan. Participation was voluntary; informed consent was taken and confidentiality assured. The study protocol was reviewed and approved by the institutional ethics committee (Approval No. 00009 IHSA/P\D-2022). Data collection was guided by extensive stakeholder engagement involving students, faculty, and mental health professionals to ensure contextual relevance and acceptability. Sociodemographic variables (e.g., age, gender, year of study, socioeconomic indicators) and lifestyle factors (e.g., sleep patterns, physical activity, academic workload, and social factors) were collected. Anxiety and depression were assessed using validated self-report instruments (GAD-7 and PHQ-9).

Following data cleaning, imputation, and encoding, the dataset was split into training (80%) and testing (20%) subsets. Separate Random Forest classification models were developed for anxiety and depression due to their capacity to model complex, non-linear relationships. Hyperparameters were optimized using cross-validation. Model performance was evaluated using accuracy, sensitivity, specificity, and AUC-ROC. Explainability was enhanced through SHapley Additive exPlanations (SHAP), enabling both global and class-specific interpretation of predictors.

**Results:**

The study reported that the prevalence of depression and anxiety was 57.8% and 46.4%, respectively. The anxiety model achieved 84.36% accuracy, while the depression model achieved 81% on the test dataset. SHAP analysis identified academic performance, sleep patterns, and physical activity as the strongest predictors of anxiety and depression, demonstrating non-linear and context-specific relationships. The anxiety model showed comparatively stronger performance, potentially reflecting differences in symptom structure and their associations with lifestyle variables.

**Conclusion:**

We concluded that explainable machine learning models based on non-stigmatizing data can effectively support mental health screening among medical students. This approach offers a scalable, ethically transparent decision-support tool to inform targeted preventive interventions, such as sleep hygiene initiatives and workload management, advancing precision mental health in resource-constrained educational settings.

